# Clinical effect of early enteral nutrition support on critically ill neonates with extracorporeal membrane oxygenation

**DOI:** 10.1186/s12887-023-04171-2

**Published:** 2023-07-13

**Authors:** Ze-Wei Lin, Ying-Ying Liu, Xiu-Hua Chen, Yi-Rong Zheng, Hua Cao, Qiang Chen

**Affiliations:** Department of Cardiac Surgery, Fujian Children’s Hospital (Fujian Branch of Shanghai Children’s Medical Center), College of Clinical Medicine for Obstetrics & Gynecology and Pediatrics, Fujian Medical University, Fuzhou, China

**Keywords:** Neonates, ECMO, Respiratory and circulatory failure, Enteral nutrition

## Abstract

**Objective:**

To investigate the feasibility and clinical outcomes of early enteral nutrition (EN) in critically ill neonates supported by extracorporeal membrane oxygenation (ECMO).

**Methods:**

We retrospectively analyzed the clinical data of 16 critically ill neonates who received ECMO support for respiratory and circulatory failure from July 2021 to December 2022 at our center. The patients were divided into two groups: the early EN group (< 24 h) and the late EN group (> 24 h). The related clinical and nutrition-related indicators between the groups were compared.

**Results:**

There was a significant difference in the time from ECMO treatment to the start of EN between the early EN group (9 patients, 56.2%) and the late EN group (7 patients, 43.8%) (*P* < 0.05). However, there were no significant differences in ECMO duration, hospitalization time, vasoactive-inotropic score (VIS), intestinal oxygen saturation, or routine stool occult blood (OB) test between the two groups (all *P* > 0.05). The incidence of complications such as intestinal obstruction, abdominal distension, diarrhea, and necrotizing enterocolitis (NEC) was slightly lower in the early EN group, but the differences were not statistically significant (all *P* > 0.05). The early EN group had a shorter time [3.6 (3.5, 5) vs. 7.5 (5.9, 8.5) d] to reach full gastrointestinal nutrition compared to the late EN group (*P *< 0.05).

**Conclusion:**

Providing early nutritional support through enteral feeding to critically ill neonates receiving ECMO treatment is both safe and practical, but close monitoring of clinical and nutritional indicators is essential.

## Introduction

Extracorporeal membrane oxygenation (ECMO) is a complex medical technique that employs a variety of established and emerging technologies to provide supportive therapy for critically ill neonates and children experiencing reversible cardiopulmonary failure [1, 2]. Although patients usually experience respiratory or hemodynamic stabilization within the first few days after ECMO treatment, providing early enteral nutrition (EN) and achieving caloric goals in pediatric patients with ECMO continue to pose significant clinical challenges. Malnutrition is a common problem among critically ill patients, particularly those receiving ECMO support, and it increases the risk of morbidity and mortality [3, 4]. Therefore, providing adequate nutrition is crucial to minimize the physiological complications of critical illness and promote recovery. Several studies have reported decreased intestinal perfusion and intestinal barrier dysfunction during ECMO support [5, 6]. Thus, early initiation of EN may increase the risk of intestinal ischemic injury or NEC. Despite these concerns, some reports confirmed that early use of EN in neonatal patients was safe and well tolerated [7–9]. Moreover, EN has been shown to improve gastrointestinal immune function, promote positive nitrogen balance, and reduce sepsis-related morbidity and costs [10, 11]. At present, there are specific nutritional guidelines for the use of neonatal ECMO [12]. However, there is still a lack of research on the optimal timing of EN initiation and the clinical efficacy of EN in critically ill neonates receiving ECMO. This study aimed to retrospectively analyze the clinical data of 16 critically ill neonates supported by ECMO due to cardiopulmonary failure and investigate the clinical efficacy of early EN in neonates requiring ECMO support.

## Methods and materials

### Patient

This study retrospectively analyzed the clinical data of 16 critically ill neonates who received ECMO support for respiratory and circulatory failure from July 2021 to December 2022. The inclusion criteria were as follows: 1. aged 1–28 days; 2. veno-arterial (VA) ECMO support for more than 48 h; and 3. after VA-ECMO support, the patient’s circulation was considered stable and able to tolerate enteral nutrition (EN). The exclusion criteria were as follows: 1. gastrointestinal perforation or active bleeding; 2. complete mechanical ileus or persistent paralytic ileus; 3. severe gastrointestinal malformations; 4. severe gastrointestinal dysfunction caused by abdominal infection; and 5. milk protein allergy. The start time of EN was determined by the clinician based on the patient’s circulatory status. According to the definition, all patients were divided into an early EN group (9 patients, EN started within 24 h) and a late EN group (7 patients, EN started after 24 h) [13]. All 16 patients received ECMO treatment in our cardiac intensive care unit (CICU). All research data were collected from the electronic medical record system and inpatient database. The study was approved by the medical ethics committee of the hospital, and all family members of the patient provided oral and written informed consent.

### Intervention method

Criteria for neonatal ECMO treatment: according to the ELSO Neonatal Respiratory Support Guidelines, the criteria must have met one of the following conditions: 1. reversible cardiopulmonary failure; 2. oxygenation index > 40 and duration > 4 h; 3. after 24 h of conventional treatment, oxygen composite index > 20; 4. severe progressive respiratory failure or combined with ventricular failure, or combined with the use of high-dose cardiovascular drugs for an extended period. The absolute contraindications for neonatal ECMO treatment were as follows: 1. grade III-IV intracranial hemorrhage; 2. severe coagulation abnormalities; 3. fatal congenital malformations; and 4. chromosomal abnormalities. The relative contraindications were as follows: 1. gestational age < 34 weeks; 2. birth weight < 2000 g; 3. mechanical ventilation time > 10 days with no improvement.

Nutritional support protocol: The nutritional regimen for the enrolled patients was based on their physical examination, gastrointestinal symptoms, daily fluid intake and output, laboratory tests, ECMO parameters, ventilator parameters, dosage and types of vasoactive drugs. A daily nutritional treatment plan was formulated and prescribed by the clinical nutritionist. EN started with the principle of “nourishing” feeding, with continuous feeding of 0.5 to 1.0 ml/kg/h of breast milk or formula via a nasogastric catheter. After 4 h of enteral feeding, the patient’s tolerance was assessed, and if tolerated, the patient’s feeding rate was increased by 1 ml/kg/h every 4 h (to a maximum of 6 ml/kg/h); if intolerance occurred during this period, the feeding rate was reduced by 50% of the current dose. Breast milk was the first choice, but if breast milk was not available, donated breast milk or deeply hydrolyzed formula milk could be used [14].

### Data collection

We collected the following data: 1. clinical data, including age, weight, sex, gestational age, etiology of ECMO support, ECMO mode, ECMO duration, vasoactive-inotropic score (VIS) before ECMO, length of CICU stay, hospitalization time, etc. 2. Nutrition-related data included EN support start time, time to reach full gastrointestinal nutrition, gastrointestinal complications (intestinal obstruction, abdominal distension, diarrhea, NEC, etc.), intestinal oxygen saturation, and stool routine occult blood (OB) test.

### Statistical method

Statistical analysis was conducted using SPSS 25.0 software, with a significance level set at 0.05. The results were considered statistically significant if the P value was less than 0.05. Quantitative data were presented as the mean ± standard deviation, and comparisons between groups were made using a two-sample t test or nonparametric rank sum test. Categorical data were presented as frequencies and percentages, and comparisons between groups were made using a chi-squared test or Fisher’s exact test.

## Result

There were 16 neonates in the study, with 12 males (75%) and 4 females (25%); the median age was 2 (1.75, 4) days, and the median weight was 3.3 (2.9, 3.7) kg. All the reasons for ECMO support were respiratory and circulatory failure, including 8 cases of primary persistent pulmonary hypertension combined with respiratory failure, 2 cases of low cardiac output syndrome after congenital heart disease, 2 cases of severe pertussis infection, 2 cases of meconium aspiration syndrome, and 3 cases of surgical repair of congenital diaphragmatic hernia. ECMO was performed in the veno-arterial (VA) mode. Combined continuous renal replacement therapy (CRRT) was administered in 8 patients (accounting for 50%). The median stay in the CICU was 20.5 (12, 28.7) days, 14 patients (87.5%) were successfully withdrawn from ECMO, and 11 patients (68.7%) survived and were discharged. The median ECMO treatment time of the 16 patients was 84 (69.5, 88.5) hours, and the median length of hospital stay was 26.5 (20.5, 29.5) d.

Seven neonates in the early EN group were successfully withdrawn from ECMO, whereas 2 patients failed to do so. Of the three nonsurviving ECMO neonates in the early EN group, the reason was intractable low cardiac output syndrome. All patients in the late EN group were successfully withdrawn from ECMO. Of the two nonsurviving ECMO neonates in the late EN group, one was for inadequate cardiac output from congenital heart surgery that caused heart failure, and the other was for refractory pulmonary hypertension and secondary heart failure. Between the two groups, age [2 (1.75, 3.25) vs. 2 (1.5, 5) d], weight [3.05 (2.99, 3.26) vs. 3.65 (3.15, 3.75) kg], gestational age [38.9 (37.7, 40.1) vs. 37.3 (36.6, 38.8) w], ECMO treatment time [79 (69, 87) vs. 84 (72, 86) h], length of CICU stay [15 (12, 22) vs. 23 (17, 38) d], length of hospital stay [26 (15, 29) vs. 25 (22, 38) d], and VIS before ECMO [53 (50, 61) vs. 55 (54, 56)] were not significantly different (all *P *> 0.05) (Table 1).

**Table 1:** General clinical characteristics of neonates in the two groups

**Item**	**Early enteral nutrition group (** ***n*** ** = 9)**	**Late enteral nutrition group (** ***n*** ** = 7)**	***P*** ** value**
**Gender(Male/Female)**	7/2	5/2	0.77
**Age (d)**	2 (1.75, 3.25)	2 (1.5, 5)	0.95
**Weight (kg)**	3.05 (2.99, 3.26)	3.65 (3.15, 3.75)	0.63
**Gestational age (w)**	38.9 (37.7, 40.1)	37.3 (36.6, 38.8)	0.24
**ECMO duration (h)**	79 (69, 87)	84 (72, 86)	0.95
**VIS score before ECMO**	53 (50, 61)	55 (54, 56)	0.70
**Length of CICU stay (d)**	15 (12, 22)	23 (17, 38)	0.45
**Length of hospital stay (d)**	26 (15, 29)	25 (22, 38)	0.83

*ECMO* extracorporeal membrane oxygenation, *CICU* cardiac intensive care unit

The two sets of EN support start times [19.1 (15.5, 21.9) vs. 33.3 (27.6, 4.5) h] were significantly different (*P* = 0.001). Intestinal oxygen saturation of the two groups [56 (54.7, 57.5) vs. 56 (54.5, 57.5) %] and stool routine OB test positivity (0/9 vs. 1/7) did not show statistically significant differences (*P* > 0.05). In the early EN group, 6 patients were breastfed, and 3 patients were fed with deeply hydrolyzed formula powder. In the late EN group, 5 patients were breastfed, and 2 patients were fed deeply hydrolyzed formula milk. Eleven patients survived at the end, and all were fed by mouth before discharge.

Among the 9 neonates treated with early EN, 1 developed abdominal distension, and the rest of the patients did not experience intestinal obstruction, abdominal distension, diarrhea, or NEC. Among the 7 neonates treated with late EN, 2 patients had abdominal distension, 1 patient had a positive fecal occult blood, and the rest of the patients did not experience intestinal obstruction, abdominal distension, diarrhea, or NEC. The incidence of intestinal obstruction, abdominal distension, diarrhea, and NEC in the early EN group was slightly lower than that in the late EN group, but there was no statistical significance (*P* > 0.05). For the full gastrointestinal nutrition time of the two groups [3.6 (3.5, 4.1) vs. 7.5 (5.9, 8.5) d], the difference was statistically significant (*P* = 0.003), as shown in Tables 2 and 3.

**Table 2:** Comparison of nutrition-related data between the two groups

**Item**	**Early enteral nutrition group (** ***n*** ** = 9)**	**Late enteral nutrition group (** ***n*** ** = 7)**	***P*** ** value**
**EN support start time (h)**	19.1 (15.5, 21.9)	33.3 (27.6, 34.5)	0.001
**Time to reach full gastrointestinal nutrition (d)**	3.6 (3.5, 4.1)	7.5 (5.9, 8.5)	0.003
**Intestinal oxygen saturation (%)**	56 (54.7, 57.5)	56 (54.5, 57.5)	0.556
**Gastrointestinal Complications**			
**Intestinal obstruction**	0(0%)	0(0%)	NS
**Abdominal distension**	1(11.1%)	2(28.5%)	0.37
**Diarrhea**	0(0%)	0(0%)	NS
**NEC**	0(0%)	0(0%)	NS
**Stool routine + OB (positive)**	0(0%)	1(14.3%)	0.27

*NEC* Necrotizing enterocolitis, *OB* occult blood test

**Table 3:** Comparison of nutrition-related data between breastfeeding group and non-breastfeeding group

**Item**	**Early enteral nutrition group (** ***n*** ** = 9)**		***P*** ** value**	**Late enteral nutrition group (** ***n*** ** = 7)**		***P*** ** value**
	**Breastfeeding group (** ***n*** ** = 4)**	**Non-breastfeeding group (** ***n*** ** = 5)**		**Breastfeeding group (** ***n*** ** = 3)**	**Non-breastfeeding group (** ***n*** ** = 4)**	
**Intestinal obstruction**	0(0%)	0(0%)	NS	0(0%)	0(0%)	NS
**Abdominal distension**	0(0%)	1(20.0%)	0.38	1(33.3%)	1(33.3%)	0.81
**Diarrhea**	0(0%)	0(0%)	NS	0(0%)	0(0%)	NS
**NEC**	0(0%)	0(0%)	NS	0(0%)	0(0%)	NS
**Stool routine + OB (positive)**	0(0%)	0(0%)	NS	0(0%)	1(25.0%)	0.34

*NEC* Necrotizing enterocolitis, *OB* occult blood test

## Discussion

Critically ill neonates supported by ECMO require optimal nutritional therapy. Malnutrition is a common issue among critically ill neonates and is linked to increased morbidity and mortality [13]. Enteral nutrition, particularly early EN within 24 h, not only provides nutritional substrates but also improves the intestinal mucosal barrier and immune function and maintains intestinal microecology [15]. Early EN is becoming increasingly accepted and applied clinically. Due to factors such as hypotension, hypoperfusion, hypoxemia, and the use of vasopressors in neonates undergoing ECMO, medical staff often delay feeding for safety reasons [16]. In the past, there was much controversy surrounding whether infants on ECMO should receive EN, with concerns that hypoxia–ischemia and damage to the integrity of the intestine could lead to bacterial translocation, sepsis, and serious adverse events, such as necrotic small bowel and colon inflammation and multiple organ failure [15]. However, research has shown that while the intestinal integrity of ECMO neonates is damaged to some extent, the introduction of EN does not lead to further deterioration [12]. Adequate macronutrient support has been shown to improve outcomes, and a large multicenter study of over 1000 mechanically ventilated children found that providing 60% of prescribed protein requirements was associated with lower mortality [16–19].

A study of 96 hospitals in the United States found that in 84.2% of these neonates, EN was begun during ECMO treatment, and in 38% of cases, feeding routines were established for neonates and children on ECMO [20]. These results suggested that starting EN during ECMO had become a common practice among physicians. To investigate the benefits and outcomes of providing early EN to critically ill neonates on ECMO, our center analyzed the clinical data of 16 neonates with ECMO for respiratory and circulatory failure. Our results showed that complications such as intestinal obstruction, abdominal distension, diarrhea, and NEC were less frequent in the early EN group than in the late EN group. Additionally, the full gastrointestinal feeding time was better in the early EN group. These findings indicated that early EN could restore gastrointestinal function faster in critically ill neonates and suggested that early EN was safe and effective for neonates with ECMO. We believe that early EN could provide essential nutrients, such as calories, protein, and trace elements, safely and effectively.

Currently, there is an ongoing debate about when to begin EN during ECMO treatment. However, early EN is the widely accepted perspective by many scholars. Piena et al. initiated enteral feeding between 3–9 days of ECMO support for neonates [12]. Scott et al. retrospectively studied the safety and tolerance of early EN in 27 adult ECMO patients and found that EN could begin within the first 24–36 h of ECMO support [21]. This study found that the starting time of EN in the early EN group was 19.1 (15.5, 21.9) hours, which was earlier than those in most studies, and it was found to be safe and effective. Based on our research results, we believe that for critically ill neonates receiving ECMO support, the starting time of EN could be even shorter. In a CICU center, seven VA-ECMO patients with severe hemodynamic disorders received EN as their sole source of nutrition, 2 patients received EN within the first 24 h, all patients received EN within 48 h, and EN was generally well tolerated. Within one week, more than 70% of the nutritional target value was achieved [9]. Therefore, with appropriate medical monitoring, early EN treatment in infantile patients with VA-ECMO is feasible and safe.

In this study, in the early EN group during neonatal ECMO treatment, minimal enteral feeding was found to be safe and effective while using multiple vasoactive drugs. Early EN in critically ill neonates typically involves minimal feeding, which can stimulate the secretion of intestinal hormones and reduce the risk of heterogeneous infections compared with TPN support. Critically ill patients often require vasopressors (such as epinephrine, norepinephrine, dopamine, and vasopressin) to maintain proper hemodynamics due to hypotension and sepsis. Most vasopressors have vasoconstrictive effects, which can potentially affect gastrointestinal motility and perfusion. However, in this study, only one patient experienced mild abdominal distension during ECMO support, which subsided quickly after defecation, suggesting that early EN was safe even when using multiple vasoactive drugs during ECMO assistance. In a large trial of critically ill shock patients who received vasopressor support, 28-day all-cause mortality did not differ between parenteral nutrition and EN [22]. However, for safety reasons, it is important to closely monitor gastrointestinal conditions in patients receiving vasoactive drugs while receiving EN treatment.

This study also had some limitations. Due to the small number of such patients in our area, we could only include patients as much as possible, so the small number of patients might affect the accuracy of the results. However, we still believe that our results have certain clinical significance. Using 24 h as a time point, such simple grouping might bias the results. Much research might still be needed for determining which time point to take as the research point. In addition, there were fewer indicators to evaluate, which might also affect the results. Finally, this was a retrospective study, and the statistical titer was not as good as that of prospective clinical studies.

## Conclusion

Early enteral nutrition is safe and effective for critically ill neonates with ECMO support and has a positive impact on their prognosis. However, close monitoring of clinical and nutritional indicators is essential.

## Data Availability

The datasets used and analyzed during the current study are available from the corresponding author at the reasonable request.

## References

[1] Ulerich L (2014). Nutrition implications and challenges of the transplant patient undergoing extracorporeal membrane oxygenation therapy. Nutr Clin Pract.

[2] Hayes D, Tobias JD, Kukreja J, Preston TJ, Yates AR, Kirkby S, Whitson BA (2013). Extracorporeal life support for acute respiratory distress syndromes. Ann Thorac Med.

[3] Vaquer S, de Haro C, Peruga P, Oliva JC, Artigas A (2017). Systematic review and meta-analysis of complications and mortality of veno-venous extracorporeal membrane oxygenation for refractory acute respiratory distress syndrome. Ann Intensive Care..

[4] Biancari F, Perrotti A, Dalén M, Guerrieri M, Fiore A, Reichart D, Dell'Aquila AM, Gatti G, Ala-Kokko T, Kinnunen EM, Tauriainen T, Chocron S, Airaksinen JKE, Ruggieri VG, Brascia D (2018). Meta-analysis of the outcome after postcardiotomy venoarterial extracorporeal membrane oxygenation in adult patients. J Cardiothorac Vasc Anesth.

[5] Koning NJ, Vonk AB, van Barneveld LJ, Beishuizen A, Atasever B, van den Brom CE, Boer C (2012). Pulsatile flow during cardiopulmonary bypass preserves postoperative microcirculatory perfusion irrespective of systemic hemodynamics. J Appl Physiol (1985).

[6] Kurundkar AR, Killingsworth CR, McIlwain RB, Timpa JG, Hartman YE, He D, Karnatak RK, Neel ML, Clancy JP, Anantharamaiah GM, Maheshwari A (2010). Extracorporeal membrane oxygenation causes loss of intestinal epithelial barrier in the newborn piglet. Pediatr Res.

[7] Jaksic T, Hull MA, Modi BP, Ching YA, George D, Compher C, American Society for Parenteral and Enteral Nutrition (A.S.P.E.N.) Board of Directors (2010) (2010). A.S.P.E.N. Clinical guidelines: nutrition support of neonates supported with extracorporeal membrane oxygenation. JPEN J Parenter Enteral Nutr..

[8] Lukas G, Davies AR, Hilton AK, Pellegrino VA, Scheinkestel CD, Ridley E (2010). Nutritional support in adult patients receiving extracorporeal membrane oxygenation. Crit Care Resusc.

[9] Umezawa Makikado LD, Flordelís Lasierra JL, Pérez-Vela JL, Montejo González JC (2013). Nutrition support during extracorporeal membrane oxygenation (ECMO) in adults. Intensive Care Med.

[10] Kreymann KG, Berger MM, Deutz NE, Hiesmayr M, Jolliet P, Kazandjiev G, Nitenberg G, van den Berghe G, Wernerman J, Ebner C, Hartl W, Heymann C, Spies C, DGEM (German Society for Nutritional Medicine), ESPEN (European Society for Parenteral and Enteral Nutrition) (2006). ESPEN Guidelines on Enteral Nutrition: Intensive care. Clin Nutr..

[11] Dellinger RP, Levy MM, Carlet JM, Bion J, Parker MM, Jaeschke R, Reinhart K, Angus DC, Brun-Buisson C, Beale R, Calandra T, Dhainaut JF, Gerlach H, Harvey M, Marini JJ, Marshall J, Ranieri M, Ramsay G, Sevransky J, Thompson BT, … Vincent JL. Surviving Sepsis Campaign: international guidelines for management of severe sepsis and septic shock: 2008. Intensive Care Med. 2008;34(1):17–60. 10.1007/s00134-007-0934-2. PMID: 18058085; PMCID: PMC2249616. 10.1007/s00134-007-0934-2 PMC2249616 18058085

[12] Piena M, Albers MJ, Van Haard PM, Gischler S, Tibboel D (1998). Introduction of enteral feeding in neonates on extracorporeal membrane oxygenation after evaluation of intestinal permeability changes. J Pediatr Surg.

[13] Bechard LJ, Duggan C, Touger-Decker R, Parrott JS, Rothpletz-Puglia P, Byham-Gray L, Heyland D, Mehta NM (2016). Nutritional status based on body mass index is associated with morbidity and mortality in mechanically ventilated critically Ill children in the PICU. Crit Care Med.

[14] Salvatori G, De Rose DU, Massolo AC, Patel N, Capolupo I, Giliberti P, Evangelisti M, Parisi P, Toscano A, Dotta A, Di Nardo G (2022). Current strategies to optimize nutrition and growth in newborns and infants with congenital heart disease: a narrative review. J Clin Med.

[15] Herbert G, Perry R, Andersen HK, Atkinson C, Penfold C, Lewis SJ, Ness AR, Thomas S (2019). Early enteral nutrition within 24 hours of lower gastrointestinal surgery versus later commencement for length of hospital stay and postoperative complications. Cochrane Database Syst Rev.

[16] Greathouse KC, Sakellaris KT, Tumin D, Katsnelson J, Tobias JD, Hayes D, Yates AR (2018). Impact of early initiation of enteral nutrition on survival during pediatric extracorporeal membrane oxygenation. JPEN J Parenter Enteral Nutr.

[17] Ohman K, Zhu H, Maizlin I, Williams RF, Guner YS, Russell RT, Harting MT, Vogel AM, Starr JP, Johnson D, Ramirez R, Manning L (2020). A multicenter study of nutritional adequacy in neonatal and pediatric extracorporeal life support. J Surg Res.

[18] Mehta NM, Bechard LJ, Zurakowski D, Duggan CP, Heyland DK (2015). Adequate enteral protein intake is inversely associated with 60-d mortality in critically ill children: a multicenter, prospective, cohort study. Am J Clin Nutr.

[19] Mehta NM, Bechard LJ, Cahill N, Wang M, Day A, Duggan CP, Heyland DK (2012). Nutritional practices and their relationship to clinical outcomes in critically ill children–an international multicenter cohort study*. Crit Care Med.

[20] Desmarais TJ, Yan Y, Keller MS, Vogel AM (2015). Enteral nutrition in neonatal and pediatric extracorporeal life support: a survey of current practice. J Pediatr Surg.

[21] Scott LK, Boudreaux K, Thaljeh F, Grier LR, Conrad SA (2004). Early enteral feedings in adults receiving venovenous extracorporeal membrane oxygenation. JPEN J Parenter Enteral Nutr.

[22] Reignier J, Boisramé-Helms J, Brisard L, Lascarrou JB, Ait Hssain A, Anguel N, Argaud L, Asehnoune K, Asfar P, Bellec F, Botoc V, Bretagnol A, Bui HN, Canet E, Da Silva D, Darmon M, Das V, Devaquet J, Djibre M, Ganster F, … Clinical Research in Intensive Care and Sepsis (CRICS) group. Enteral versus parenteral early nutrition in ventilated adults with shock: a randomised, controlled, multicentre, open-label, parallel-group study (NUTRIREA-2). Lancet. 2018;391(10116):133–143. 10.1016/S0140-6736(17)32146-3. PMID: 29128300. 10.1016/S0140-6736(17)32146-3 29128300

